# Prognostic significance of tumor deposit counts in stage III colorectal cancer based on T/N staging and chemotherapy status: A retrospective cohort study

**DOI:** 10.1016/j.sopen.2025.02.004

**Published:** 2025-02-22

**Authors:** Chenxiao Zheng, Lingsha Xu, Binbin Ou, Ibrahim Mohamed Bakour Abdourahaman, Xuanqin Chen, Hangjia Xu, Yating Zheng, Yifei Pan

**Affiliations:** a The First Affiliated Hospital of Wenzhou Medical University, PR China; b Gongshu District Integrated Traditional Chinese and Western Medicine Hospital in Hangzhou, PR China; c The First Affiliated Hospital of Wenzhou Medical University, Department of Colorectal Anal Surgery, PR China

**Keywords:** Tumor deposit, Stage III, Colorectal cancer, Adjuvant chemotherapy, Prognosis

## Abstract

**Background:**

We aimed to evaluate the impact of tumor deposit (TD) count on cancer-specific survival (CSS) and disease-free survival (DFS) in stage III colorectal cancer (CRC) patients stratified by T and N staging, and further explore its impact on chemotherapy effect.

**Method:**

We determined the optimal TD cut-off value for stage III CRC patients from the SEER database utilizing X-tile analysis, and retrospectively analyzed the clinicopathological data of 443 patients from the First Affiliated Hospital of Wenzhou Medical University from 2019 to 2020. Chi-square (χ2) tests compared categorical variables. Kaplan–Meier assessed CSS and DFS. Cox regression model evaluated prognostic factors on CSS and DFS.

**Results:**

2TD is the optimal cutoff value for prognosis in Stage III CRC, in the low-risk group (T1-T3 and N1), ≥3TD patients faced higher cancer-specific mortality (HR = 3.445, 95%CI = 1.254–9.465, P = 0.017) and recurrence risks (HR = 1.934, 95%CI = 1.095–3.416, P = 0.024) vs. 1-2TD, while 1-2TD and no-TD patients showed no difference in survival. In the high-risk group (T4 or N2), both ≥3TD and 1-2TD patients had poor prognosis. Chemotherapy reduced cancer-specific mortality in both groups (1-2TD: HR = 0.347, 95%CI = 0.138–0.870, P = 0.024; ≥3TD: HR = 0.272, 95%CI = 0.077–0.960, P = 0.043) but did not significantly improve recurrence risk (1-2TD: P = 0.177; ≥3TD: P = 0.058).

**Conclusion:**

TD indicates poor prognosis in stage III CRC, with ≥3 TD significantly worsening survival, yet the prognosis remains poor in TD-positive patients with high-risk (T4 or N2) regardless of TD count. Moreover, TD count does not influence chemotherapy's mortality benefit.

## Introduction

Colorectal cancer (CRC) ranks third globally in cancer prevalence and second in cancer deaths [1]. China reports high CRC incidence, with 555,477 new cases and 286,162 deaths in 2020. Many patients are diagnosed in advanced stages, with 1/3 exhibiting lymph node metastasis (stage III CRC). Recurrence rates reach 30 % in these patients [2]. TNM staging, crucial for prognosis and chemotherapy strategies, recommends adjuvant chemotherapy for all stage III CRC patients [3]. However, TNM staging lacks comprehensive prognostic information, resulting in survival differences even within the same stage, particularly in stage III CRC [4]. Therefore, risk stratification and prognostic markers are crucial for accurate prognosis in stage III patients. In the 2017 8th edition of the UICC/AJCC staging system, tumor deposit (TD) is defined as cancerous nodules distant from the primary tumor, within the lymphatic drainage area of the colorectal region, without any recognizable residual lymph node, vessel, or nerve structures [4]. Multiple studies link TD to poor prognosis [5], hence, it was categorized as pN1c in the 7th edition TNM classification (TNM7). In TNM8, TD with histological features of venous, lymphatic, or perineural invasion are classified accordingly, and no longer considered TD. However, TD doesn't affect tumor staging in cases of lymph node metastasis (LNM), sparking debate due to its limited prognostic value [6].

Research demonstrates that TD are independent prognostic indicators for postoperative CRC patients, regardless of lymph node status, and are associated with poorer outcomes [[7], [8], [9]].Concurrently, TD count offers supplementary prognostic info for CRC patients. Multiple studies propose including TD in LNM count or integrating them into the next TNM system [[10], [11], [12], [13]]. Nevertheless, the prognostic discriminatory ability of these revised systems remains to be assessed.

Adjuvant chemotherapy enhances DFS for stage III CRC patients [14], but its potential toxicity necessitates risk-based treatment strategies. The IDEA study pioneered risk stratification for stage III colon cancer, demonstrating non-inferiority of shortened oxaliplatin-based chemotherapy [15]. The difference in DFS between low-risk and high-risk patients was 20 % (3-year DFS of 83 % vs. 64 %). Given the rationality of risk stratification and the potential underestimation of prognosis caused by ignoring the number of TD, and based on prior research on TD, we hypothesize TD has greater prognostic significance in low-risk (T1–3 and N1) than high-risk (T4 or N2) stage III CRC patients. We aim to determine the optimal TD cutoff using X-tile and assess its prognostic influence across T and N stage stratifications. Additionally, we further explored the impact of TD counts on survival after chemotherapy.

## Patients and methods

### Patients

**Fig. 1:**
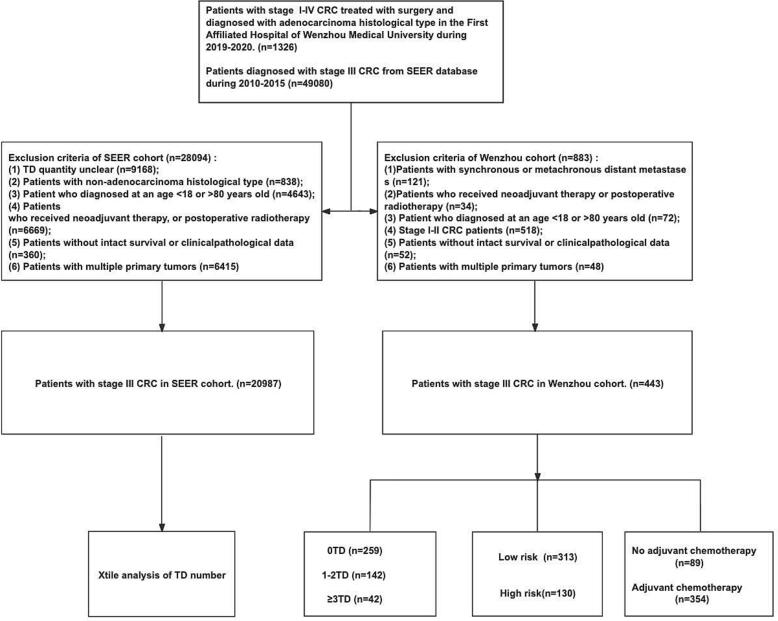
Flow chart of the selection process for the study.

This retrospective cohort study included 443 cases that met the research criteria out of 1326 patients with stage III CRC who underwent radical resection at the First Affiliated Hospital of Wenzhou Medical University from January 1, 2019, to December 30, 2020. We excluded 883 patients who met the following exclusion criteria: Patients with synchronous or metachronous distant metastasis (n = 121); patients with other primary malignancies or multiple colorectal malignancies (n = 48); patients with a diagnosis age below 18 years or above 80 years (n = 72); patients with non-stage III CRC (n = 518); patients who received neoadjuvant therapy, or postoperative radiotherapy (n = 34); patients with missing important clinicopathological data (n = 52). Eligible patients were categorized into surgery-only (n = 87) and adjuvant chemotherapy groups (n = 356) as well as low-risk (T1-T3, N1, n = 313) and high-risk (T4 or N2, n = 130) groups. Meanwhile, the X-tile analysis determined the optimal cutoff value for the TD number among 20,987 stage III CRC patients diagnosed between 2010 and 2015 from the SEER^∗^Stat software Version 8.4.3, the selection process of two cohort is illustrated in Fig. 1. Accordingly, local patients were divided into three groups (0TD,1-2TD,≥3TD). All patients were staged according to the American Joint Committee on Cancer (AJCC) 7th edition TNM staging system. Kaplan-Meier, COX regression, and subgroup analyses were conducted to assess differences in CSS and DFS among these groups.

### Data and follow up

The clinicopathological and survival information of the SEER cohort are from SEER∗Stat software Version 8.4.3. The demographic and clinicopathological characteristics of local cohort were obtained from the database of hospital, including: age at diagnosis (year), gender (male or female), tumor location (right colon: cecum to transverse colon; left colon: descending colon and sigmoid colon; rectum), tumor size, tumor differentiation degree (low differentiation, moderate differentiation, high differentiation), histological type (adenocarcinoma), morphological type (ulcerative type, expansive type, infiltrative type), the receipt of chemotherapy (no or yes), vascular invasion, perineural invasion, TD count (0TD, 1–2 TD, TD ≥ 3), lymph nodes examined (<12 or ≥12), T stage (T1,T2,T3,T4), N stage (N1a,N1b,N1c,N2a,N2b), risk stratification (low risk: T1-T3 and N1 patients, high risk: T4 or N2 patients). We routinely report the number of TD and LNM in all pathological reports of radical surgery for colorectal cancer. All our pathological reports are cross-diagnosed by two senior pathologists and reviewed by a third. For the hospital cohort, postoperative complications data were collected. Complications of grade II or higher according to the 2009 modified Clavien-Dindo classification system were defined as clinically significant. Follow-up data were collected from the colorectal surgery follow-up database. The primary endpoints were CSS and DFS. Patients alive on October 1, 2023, were considered as censored cases.

#### Adjuvant chemotherapy

According to the NCCN guidelines for the treatment of CRC, all stage III patients are recommended to receive 4–8 cycles of adjuvant chemotherapy (mainly CAPEOX and FOLFOX) after surgery. However, the adjuvant chemotherapy regimen may be modified or cancelled due to age, comorbidities, or patient preference. The patients were divided into three treatment groups: surgery alone (n = 89), 5-FU monotherapy chemotherapy (n = 44), and oxaliplatin-based chemotherapy (n = 310). The adjuvant treatment should be started within 2 months after surgery and should be administered for 6 months (Stage III CRC patients with low risk may consider CAPEOX regimen for 3 months). In our cohort, the average interval from surgery to adjuvant chemotherapy (AC) was 28.5 days (range: 10–105 days). Of 354 patients receiving AC, 275 (77.6 %) completed all courses, the completion rate is 43.1 % in 5-FU monotherapy (≥8 cycles), 64.7 % in CAPEOX (4–8 cycles), and 66.6 % in FOLFOX (≥12 cycles).

#### Postoperative follow-up

All patients underwent postoperative follow-up, which adhered to the CSCO guidelines for CRC version 2023 [16] and conducted through regular outpatient, inpatient re-examination or telephone return visit. The follow-up frequency is once every 3 months for a total of 3 years, followed by once every 6 months until 5 years after surgery. The follow-up procedures include physical examination, CEA level measurement, an annual chest, abdomen, and pelvis CT scan, and colonoscopy when necessary. The follow-up was terminated when the patient died, and the final follow-up cutoff date was October 1, 2023.

## Statistical analysis

Categorical variables were expressed as frequencies and percentages, and continuous variables were expressed as medians and ranges. Pearson χ2 test was used to analyze the differences in the distribution of categorical variables among stage III CRC patients grouped by risk stratification and TD count. X-tile software (Yale University, version 3.6.1) was used to determine the optimal cut-off value of continuous variables in survival prognosis. DFS and CSS were measured by the time interval between the date of radical resection and the date of recurrence or metastasis, cancer-related death, or last follow-up. Kaplan-Meier method was used to evaluate CSS and DFS. Log Rank test evaluated the significance of differences in CSS or DFS between subgroups. Cox regression model calculated the hazard ratio (HR) and 95 % confidence interval (CI) to assess the prognostic value of various variables on DFS and CSS. All statistical analyses were performed using IBM SPSS Statistics 26.0 (IBM, Armonk, NY, USA), P value < 0.05 was considered statistically significant.

## Results

### Baseline characteristics

**Fig. 2:**
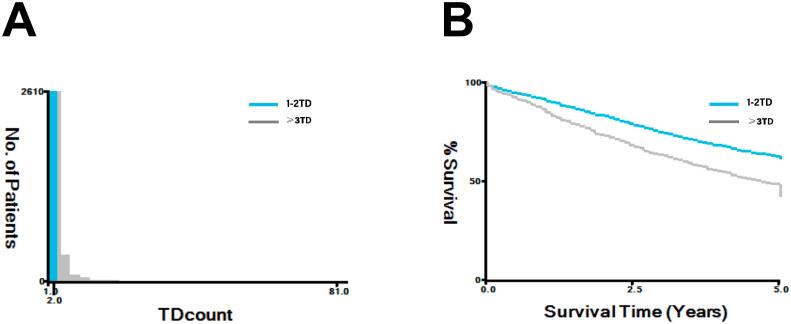
X-tile analysis determines the optimal cut-off value for TD identified according to CSS. (A) The optimal cut-off value was identified as 2; (B) The CSS rate between the two groups.

Our study encompassed 443 stage III CRC patients, with 275 (62.1 %) males and 168 (37.9 %) females. Mean age at diagnosis was 64.68 years, with 215 (48.5 %) ≤65 and 228 (51.5 %) >65. TD status revealed 259 (58.5 %) TD-negative and 184 (41.5 %) TD-positive patients. The X-tile analysis of SEER cohort determined that 2TD was the optimal cut-off value to predict CSS (Fig. 2). Thus, our cohort were divided into three groups in the subsequent analysis, including 142 (32.1 %) with 1-2TD and 42 (9.4 %) with ≥3TD. Risk stratification indicated 313 (70.6 %) low-risk and 130 (29.3 %) high-risk patients. T staging revealed 11 (2.5 %) T1, 77 (17.4 %) T2, 334 (75.4 %) T3, and 21 (4.7 %) T4 patients. N staging showed 329 (74.2 %) N1 and 114 (25.7 %) N2 patients. Treatments included 89 (20.0 %) surgery alone, 44 (9.9 %) fluorouracil monotherapy, and 310 (69.9 %) oxaliplatin-based chemotherapy. Overall, 69 patients (15.6 %) experienced clinically significant complications, including anastomotic leakage in 20 cases (4.51 %), intestinal obstruction in 19 cases (4.28 %), pulmonary infection in 15 cases (3.38 %), stoma-related complications in 5 cases (5.95 %), postoperative bleeding in 12 cases (1.12 %), abdominal/pelvic infection in 8 cases (1.80 %), urinary retention/dysuria/urinary tract infection in 8 cases (1.80 %), wound infection in 6 cases (1.35 %), and venous thrombosis in 2 cases (0.45 %).No difference was observed in postoperative complications between patients who received and did not receive AC (P = 0.305). In terms of survival, 119 (26.8 %) patients had tumor recurrence, and 54 (12.2 %) died of cancer-related causes. Baseline characteristics are summarized in Table 1.

**Table 1:** Baseline characteristics of all patients.

Characteristic	Overall (N = 443)	TD-negative (N = 259)	TD-positive (N = 184)	P-value⁎
**Age**				0.047
≤65	215 (48.5 %)	136 (52.5 %)	79 (42.9 %)	
>65	228 (51.5 %)	123 (47.5 %)	105 (57.1 %)	
**Gender**				0.042
Male	275 (62.1 %)	171 (66.0 %)	104 (56.5 %)	
Female	168 (37.9 %)	88 (34.0 %)	80 (43.5 %)	
**Primary site**				0.195
Right colon	72 (16.3 %)	49 (18.9 %)	23 (12.5 %)	
Left colon	82 (18.5 %)	46 (17.8 %)	36 (19.6 %)	
Rectum	289 (65.2 %)	164 (63.3 %)	125 (67.9 %)	
**Tumor size**				0.182
≦4 cm	295 (66.6 %)	179 (69.1 %)	116 (63.0 %)	
>4 cm	148 (33.4 %)	80 (30.9 %)	68 (37.0 %)	
**Tumor differentiation**				0.334
Poor	49 (11.1 %)	30 (11.6 %)	19 (10.3 %)	
Moderate	324 (73.1 %)	183 (70.7 %)	141 (76.6 %)	
Well	70 (15.8 %)	46 (17.8 %)	24 (13.0 %)	
**Morphological type**				0.226
Ulcerative	328 (74.0 %)	195 (75.3 %)	133 (72.3 %)	
Expansive	97 (21.9 %)	57 (22.0 %)	40 (21.7 %)	
Infiltrative	18 (4.1 %)	7 (2.7 %)	11 (6.0 %)	
**T stage**				<0.001
1	11 (2.5 %)	10 (3.9 %)	1 (0.5 %)	
2	77 (17.4 %)	60 (23.2 %)	17 (9.2 %)	
3	334 (75.4 %)	183 (70.7 %)	151 (82.1 %)	
4	21 (4.7 %)	6 (2.3 %)	15 (8.2 %)	
**N stage**				<0.001
1a	144 (32.5 %)	105 (40.5 %)	39 (21.2 %)	
1b	123 (27.8 %)	78 (30.1 %)	45 (24.5 %)	
1c	62 (14 %)	0 (0 %)	62 (33.7 %)	
2a	70 (15.8 %)	48 (18.5 %)	22 (12.0 %)	
2b	44 (9.9 %)	28 (10.8 %)	16 (8.7 %)	
**Risk classification**				0.290
Low risk	313 (70.7 %)	178 (68.7 %)	135 (73.4 %)	
High risk	130 (29.3 %)	81 (31.3 %)	49 (26.6 %)	
**Lymph node yield**				0.007
<12	95 (21.4 %)	44 (17.0 %)	51 (27.7 %)	
≧12	348 (78.6 %)	215 (83.0 %)	133 (72.3 %)	
**Vascular invasion**				0.433
Negative	182 (42.0 %)	107 (43.2 %)	75 (40.2 %)	
Positive	247 (55.8 %)	121 (55.2 %)	104(56.6 %)	
Unknown	10 (2.3 %)	4 (1.5 %)	6 (3.3 %)	
**Perineural invasion**				0.041
Negative	185 (41.8 %)	120 (46.3 %)	65 (35.3 %)	
Positive	248 (56.0 %)	135 (52.1 %)	113 (61.4 %)	
Unknown	10 (2.3 %)	4 (1.5 %)	6 (3.3 %)	
**Chemotherapy**				0.147
Yes	354(79.9 %)	213 (82.2 %)	141 (76.6 %)	
No	89 (20.1 %)	46 (17.8 %)	43 (23.4 %)	
**Preoperative CEA**				0.012
Negative	255 (57.6 %)	162 (62.5 %)	93 (50.5 %)	
Positive	188 (42.4 %)	97 (37.5 %)	91 (49.5 %)	
**Preoperative CA199**				0.367
Negative	381 (86.0 %)	226 (87.3 %)	155 (84.2 %)	
Positive	62 (14.0 %)	33 (12.7 %)	29 (15.8 %)	
Regime				0.076
CAPE	44 (9.9 %)	26 (10.0 %)	18 (9.8 %)	
CAPEOX	295 (66.6 %)	182 (70.3 %)	113 (61.4 %)	
FOLFOX	15 (3.4 %)	5 (1.9 %)	10 (5.4 %)	
No	89 (20.1 %)	46 (17.8 %)	43 (23.4 %)	
**Significant complication**				0.215
No	374 (84.42)	214 (82.63)	160 (86.96)	
Yes	69 (15.58)	45 (17.37)	24 (13.04)	

⁎ Pearson's Chi-squared test. The P-value for significance was <0.05.

It revealed that TD association with higher age (P = 0.047), male gender (P = 0.042), worse T & N staging (P < 0.001), fewer lymph nodes yield (<12) (P = 0.007), perineural invasion (P = 0.012), and higher CEA (P = 0.012). TD-positive and TD-negative groups showed no significant differences in tumor location, tumor size, differentiation grade, morphological type, risk stratification, vascular invasion, chemotherapy status, preoperative CA19-9 level, or postoperative complications (P > 0.05). Characteristics of patients stratified by risk level and presence of TD are shown in Table 2.

**Table 2:** Comparison of clinicopathological features between TD positive and negative groups stratified by risk level.

	Low risk (N = 313)				High risk (N = 130)			
Characteristic	Overall (N = 313)	TD-negative (N = 178)	TD-positive (N = 135)	P-value⁎	Overall (N = 130)	TD-negative (N = 81)	TD-positive (N = 49)	P-value⁎
**Age**				0.017				0.894
≤65	147 (47.0 %)	94 (53 %)	53 (39.3 %)		68 (52.3 %)	42 (51.9 %)	26 (53.1 %)	
>65	166 (53.0 %)	84 (47 %)	82 (60.7 %)		62 (47.7 %)	39 (48.1 %)	23 (46.9 %)	
**Gender**				0.123				0.162
Male	196 (62.6 %)	118 (66.3 %)	78 (57.8 %)		79 (60.8 %)	53 (65.4 %)	26 (53.1 %)	
Female	117 (37.4 %)	60 (33.7 %)	57 (42.2 %)		51 (39.2 %)	28 (34.6 %)	23 (46.9 %)	
**Primary site**				0.048				0.536
Right colon	53 (16.9 %)	38 (21.3 %)	15 (11.1 %)		19 (14.6 %)	11 (13.6 %)	8 (16.3 %)	
Left colon	63 (20.1 %)	36 (20.2 %)	27 (20.0 %)		19 (14.6 %)	10 (12.3 %)	9 (18.4 %)	
Rectum	197 (62.9 %)	104 (58.4 %)	93 (68.9 %)		92 (70.8 %)	60 (74.1 %)	32 (65.3 %)	
**Tumor size**				0.144				0.802
≦4 cm	211 (67.4 %)	126 (70.8 %)	85 (63.0 %)		84 (64.6 %)	53 (65.4 %)	31 (63.3 %)	
>4 cm	102 (32.6 %)	52 (29.2 %)	50 (37.0 %)		46 (35.4 %)	28 (34.6 %)	18 (36.7 %)	
**Tumor differentiation**				0.377				0.699
Poor	24 (7.7 %)	15 (8.4 %)	9 (6.7 %)		25 (19.2 %)	15 (18.5 %)	10 (20.4 %)	
Moderate	231 (73.8 %)	126 (70.8 %)	105 (77.8 %)		93 (71.5 %)	57 (70.4 %)	36 (73.5 %)	
Well	58 (18.5 %)	37 (20.8 %)	21 (15.6 %)		12 (9.2 %)	9 (11.1 %)	3 (6.1 %)	
**Morphological type**				0.545				0.367
Ulcerative	232 (74.1 %)	134 (75.3 %)	98 (72.6 %)		96 (73.8 %)	61 (75.3 %)	35 (71.4 %)	
Expansive	71 (22.7 %)	40 (22.5 %)	31 (23.0 %)		26 (20.0 %)	17 (21.0 %)	9 (18.4 %)	
Infiltrative	10 (3.2 %)	4 (2.2 %)	6 (4.4 %)		8 (6.2 %)	3 (3.7 %)	5 (10.2 %)	
**T stage**				<0.001				<0.001
1	10 (3.2 %)	9 (5.1 %)	1 (0.7 %)		1 (0.8 %)	1 (1.2 %)	0 (0.0 %)	
2	55 (17.6 %)	41 (23.0 %)	14 (10.4 %)		22 (16.9 %)	19 (23.5 %)	3 (6.1 %)	
3	248 (79.2 %)	128 (71.9 %)	120 (88.9 %)		86 (66.2 %)	55 (67.9 %)	31 (63.3 %)	
4					21 (16.2 %)	6 (7.4 %)	15 (30.6 %)	
**N stage**				<0.001				0.016
1a	139 (44.4 %)	102 (57.3 %)	37 (27.4 %)		5 (3.8 %)	3 (3.7 %)	2 (4.1 %)	
1b	117 (37.4 %)	76 (42.7 %)	41 (30.4 %)		6 (4.6 %)	2 (2.5 %)	4 (8.2 %)	
1c	57 (18.2 %)	0 (0.0 %)	57 (42.2 %)		5 (3.8 %)	0 (0 %)	5 (10.2 %)	
2a					70 (53.8 %)	48 (59.3 %)	22 (44.9 %)	
2b					44 (33.8 %)	28 (34.6 %)	16 (32.7 %)	
**Lymph node yield**				0.014				0.393
<12	78 (24.9 %)	35 (19.7 %)	43 (31.9 %)		17 (13.1 %)	9 (11.1 %)	8 (16.3 %)	
≧12	235 (75.1 %)	143 (80.3 %)	92 (68.1 %)		113 (86.9 %)	72 (88.9 %)	41 (83.7 %)	
**Vascular invasion**				0.377				0.087
Negative	133 (42.5 %)	73 (41.0 %)	60 (44.4 %)		53 (40.8 %)	39 (48.1 %)	13 (27 %)	
Positive	174 (55.6 %)	103 (57.9 %)	71 (52.6 %)		73 (56.2 %)	40 (49.4 %)	33 (67 %)	
Unknown	6 (1.9 %)	2 (1.1 %)	4 (3.0 %)		4 (3.1 %)	2 (2.5 %)	3 (6.1 %)	
**Perineural invasion**				0.366				0.005
Negative	148 (47.3 %)	90 (50.6 %)	58 (43 %)		37 (28.5 %)	30 (37.0 %)	7 (14.3 %)	
Positive	159 (50.8 %)	85 (47.8 %)	74 (55 %)		89 (68.5 %)	50 (61.7 %)	39 (79.6 %)	
Unknown	6 (1.9 %)	3 (1.7 %)	3 (2.2 %)		4 (3.1 %)	1 (1.2 %)	3 (6.1 %)	
**Adjuvant chemotherapy**				0.177				0.524
No	61 (19.5 %)	30 (16.9 %)	31 (23.0 %)		28 (21.5 %)	16 (19.8 %)	12 (24.5 %)	
Yes	252 (80.5 %)	148 (83.1 %)	104 (77.0 %)		102 (78.5 %)	65 (80.2 %)	37 (75.5 %)	
**Preoperative CEA**				0.266				0.002
Negative	185 (59.1 %)	110 (61.8 %)	75 (55.6 %)		70 (53.8 %)	52 (64.2 %)	18 (36.7 %)	
Positive	128 (40.9 %)	68 (38.2 %)	60 (44.4 %)		60 (46.2 %)	29 (35.8 %)	31 (63.3 %)	
**Preoperative CA19-9**				0.767				0.217
Negative	271 (86.6 %)	155 (87.1 %)	116 (85.9 %)		110 (84.6 %)	71 (87.7 %)	39 (79.6 %)	
Positive	42 (13.4 %)	23 (12.9 %)	19 (14.1 %)		20 (15.4 %)	10 (12.3 %)	10 (20.4 %)	
**Regime**				0.082				0.838
CAPE	32 (10.2 %)	18 (10.1 %)	14 (10.4 %)		12 (9.2 %)	8 (9.9 %)	4 (8.2 %)	
CAPEOX	209 (66.8 %)	127 (71.4 %)	82 (60.8 %)		86 (66.2 %)	55 (67.9 %)	31 (63.3 %)	
FOLFOX	11 (3.5 %)	3 (1.7 %)	8 (5.9 %)		4 (3.1 %)	2 (2.5 %)	2 (4.1 %)	
No	61 (19.5 %)	30 (16.8 %)	31 (22.9 %)		28 (21.5 %)	16 (19.8 %)	12 (24.5 %)	
**Significant complication**				0.787				0.082
No	267 (85.3 %)	151 (84.8 %)	116 (85.9 %)		107 (82.3 %)	63 (77.8 %)	44 (89.8 %)	
Yes	46 (14.7 %)	27 (15.2 %)	19 (14.1 %)		23 (17.7 %)	18 (22.2 %)	5 (10.2 %)	

⁎ Pearson's Chi-squared test. The P-value for significance was <0.05.

We found that TD correlates with poorer T & N staging (P < 0.05) in both risk groups. In low-risk group, TD is linked to older age (P = 0.017), specific tumor location (rectum > left colon > right colon) (P = 0.017), and fewer lymph nodes yield (P = 0.014). However, in the high-risk group, TD is associated with perineural invasion (P = 0.005) and higher CEA (P = 0.002).

### Kaplan-Meier survival analyses

Kaplan-Meier curves of CSS and DFS for stage III rectal cancer patients in local cohort stratified by risk level are shown in Supplementary Fig. 1. The significant differences between low-risk and high-risk groups (3-year CSS: 92.8 % vs 89.1 %, P = 0.023, Supplementary Fig. 1A; 3-year DFS: 85.3 % vs 77.2 %, P = 0.023, Supplementary Fig. 1B) demonstrated the rationality of including rectal cancer patients in our stratification method.

**Fig. 3:**
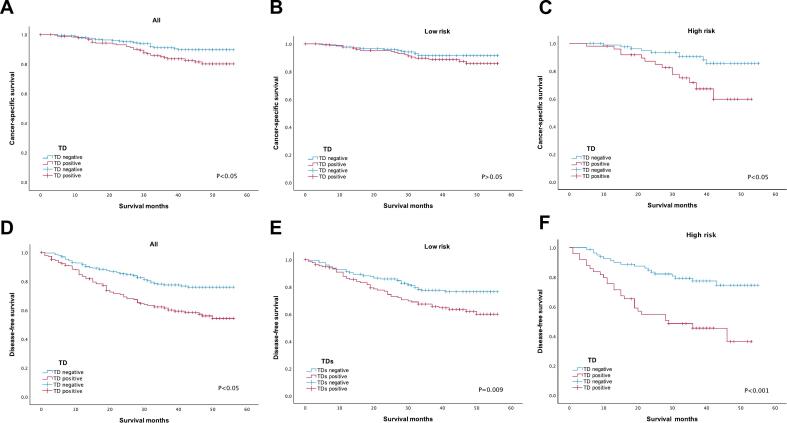
Survival curves for stage III CRC patients, stratified by TD status and risk level: (A) CSS of all patients; (B) CSS of low-risk patients; (C) CSS of high-risk patients; (D) DFS of all patients; (E) DFS of low-risk patients; (F) DFS of high-risk patients.

Kaplan-Meier curves of CSS and DFS, stratified by TD status, reveal prognostic disparities in stage III CRC patients with varying risks. In Fig. 3, TD-positive patients showed significantly poorer CSS (P = 0.02, Fig. 3A) and DFS (P < 0.001, Fig. 3D) compared to TD-negative patients, indicating the negative impact of TD on survival. Among low-risk patients, CSS differences were non-significant (P = 0.249, Fig. 3B), while DFS was worse in the TD-positive group (P = 0.009, Fig. 3E). Conversely, in high-risk patients, both CSS (P < 0.05, Fig. 3C) and DFS (P < 0.001, Fig. 3F) differed significantly between TD-negative and TD-positive groups.

**Fig. 4:**
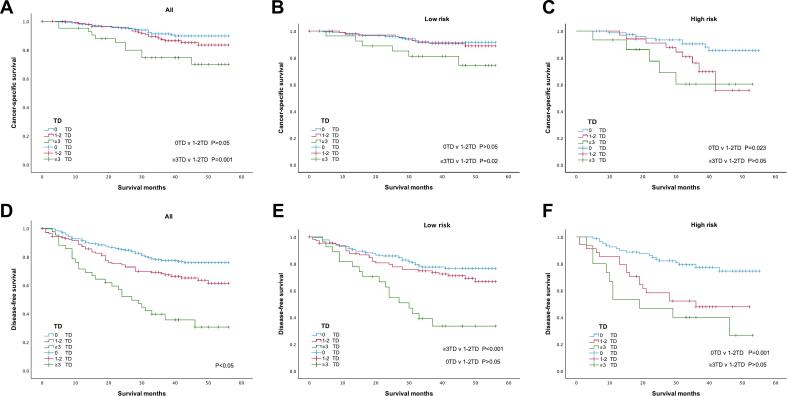
Survival curves for stage III CRC patients, stratified by TD count and risk level: (A) CSS of all patients; (B) CSS of low-risk patients; (C) CSS of high-risk patients; (D) DFS of all patients; (E) DFS of low-risk patients; (F) DFS of high-risk patients.

Fig. 4 displays Kaplan-Meier curves of CSS and DFS, calculated based on TD counts. Among all patients, those with ≥3TD fare worse in CSS (P = 0.001, Fig. 4A) and DFS (P < 0.001, Fig. 4D) than those with 1-2TD. In low-risk patients, CSS and DFS are similar between 1-2TD and 0TD patients (P > 0.05, Fig. 4B, E), while ≥3TD patients fare worse (P = 0.02, Fig. 4B; P < 0.001, Fig. 4E) vs. 1-2TD. Conversely, in high-risk patients, there is no significant difference in CSS and DFS between ≥3TD and 1-2TD patients, but both fare worse than no-TD patients (P < 0.05, Fig. 4C, F).

### Multivariate Cox analyses

We validated the results using multivariate Cox regression models, incorporating variables from univariate analysis. The results of multivariate COX analysis of all covariates selected by univariate COX analysis in subgroups of risks and TD counts are shown in Supplementary Tables 1–5. Table 3 and Supplementary Table 5 integrate the results of the multivariate COX analysis for different TD counts, it reveals that among low-risk patients, ≥3TD increase cancer-specific mortality (HR = 3.445, 95%CI = 1.254–9.465, P = 0.017; Supplementary Table 5) and recurrence risk (HR = 1.934, 95%CI = 1.095–3.416, P = 0.024; Supplementary Table 5) vs.1-2TD. In addition, 1-2TD do not significantly increase survival risk vs. no-TD (CSS: P = 0.346; DFS: P = 0.464; Table 3). However, in high-risk group, no significant survival risk difference was observed between 1-2TD and ≥3TD patients (CSS: P = 0.466; DFS: P = 0.260; Supplementary Table 5).

**Table 3:** Multivariate Cox analysis of the CSS and DFS in stage III CRC patients with different risk levels and TD count (0TD group as reference).

		All		Low risk		High risk	
Survival	TD	HR(95%CI)	P	HR(95%CI)	P	HR(95%CI)	P
CSS	0 TD	1		1		1	
	1–2 TD	1.394 (0.803–2.154)	0.618	0.853 (0.337–2.216)	0.346	1.826 (0.494–6.663)	0.369
	≥3 TD	2.351 (1.106–4.996)	0.006	2.894 (1.291–6.526)	0.037	3.186 (1.356–7.494)	0.017
DFS	0 TD	1		1		1	
	1–2 TD	1.874 (1.031–3.416)	0.045	1.225 (0.726–2.095)	0.464	3.264 (1.143–9.337)	0.027
	≥3 TD	1.823(1.110–2.996)	0.018	2.184 (1.223–3.908)	0.008	2.534 (1.065–6.067)	0.037

To assess the prognostic impact of TD count on post-chemotherapy survival, we segregated patients based on chemotherapy administration. Multivariate Cox regression analysis indicated that adjuvant chemotherapy significantly reduced cancer-specific mortality (HR = 0.365, 95%CI = 0.205–0.651, P = 0.001; Supplementary Table 3) and recurrence risks (HR = 0.658, 95%CI = 0.436–0.995, P = 0.047; Supplementary Table 4) across all stage III CRC patients. Among TD-positive patients, both the 1-2TD (HR = 0.347, 95%CI = 0.138–0.870, P = 0.024; Table 4) and ≥3TD groups (HR = 0.272, 95%CI = 0.077–0.960, P = 0.043; Table 4) exhibited reduced cancer-specific mortality risks after chemotherapy. However, neither group showed a significant benefit in terms of recurrence risk (1-2TD: P = 0.177; ≥3TD: P = 0.058; Table 4).

**Table 4:** Multivariate Cox analysis of the CSS and DFS in stage III CRC patients with varying TD counts and chemotherapy status (non-chemotherapy group as reference).

		0TD		1–2 TD		≥3 TD	
Survival	Chemotherapy	HR(95%CI)	P	HR(95%CI)	P	HR(95%CI)	P
CSS	No	1		1		1	
	Yes	0.350(0.146–0.837)	0.018	0.347(0.138–0.870)	0.024	0.272(0.077–0.960)	0.043
DFS	No	1		1		1	
	Yes	0.434(0.211–0.891)	0.023	0.638(0.332–1.224)	0.177	0.422(0.173–1.031)	0.058

## Discussion

The discovery of TD was initially reported by Gabriel et al. in 1935 [17]. Since then, their definition and staging methods have evolved, yet their formation remains enigmatic. Goldstein and Turner's research [18] revealed that TD originates from three sources: perineural, perivascular, and intravascular, with 53 % of patients exhibiting a mix of two. Additionally, Ratto et al. [19] identified TD as having intravascular, endolymphatic, perineural, or “isolated” origins, with 59 % of cases exhibiting a single component. Other studies have also demonstrated the diverse origins of TD [20]. The anatomical invasion routes are often parallel, possibly explaining the multiple component structures in TD and contributing to the pathological features observed in TD-positive patients, including lymph node metastasis (LNM), vascular invasion, and perineural invasion [[21], [22], [23]].

IDEA France study [13] and the CALGB/SWOG 80702 Phase III trial [24] show TD negatively affects prognosis, regardless of N substage. Other retrospective studies indicate worse prognosis with increasing TD number. Goldstein et al. [18] showed that the 5-year DFS rates for patients with 1, 2, or ≥3TD were 35 %, 24 %, and 2 %, respectively (p < 0.01). Jin et al. [25] reported that the survival time of pN1c patients with ≥3TD was shorter. Pricolo et al. [26] analyzed the NCDB database, revealing pN1c patients with ≥3TD had poorer survival than those with 1–2 TD (5-year OS: 51.4 % vs. 60.6 %), resembling pN2 patient survival. Shi et al. [28] also found significant differences in CSS among patients without varying TD counts. Our study identified the optimal cut-off value of TD count in the prognosis of stage III CRC patients, and accordingly classified patients into three groups, including 0TD, 1–2TD and ≥3TD groups, the results showed increased cancer-specific mortality (HR = 2.351, 95%CI = 1.106–4.996, P = 0.026; Table 3) and recurrence risks (HR = 1.823, 95%CI = 1.110–2.996, P = 0.018; Table 3) in ≥3TD patients vs. 1-2TD. Unfortunately, no specific information on local recurrence or tumor metastasis was provided in the NCDB and SEER databases, so DFS could not be obtained, therefore, we did not include the SEER cohort in subsequent analyses.

184 TD-positive cases were encompassed in our study, constituting 41.5 % of stage III CRC patients, surpassing previous research [5], the results indicate that TD correlates with older age and advanced T/N stages (P < 0.05), likely due to late diagnosis in elderly patients, with worse T and N stage, indicating deeper invasion and LNM which promoting nerve, lymphatic, and vascular invasion, providing more invasion routes for TD. Our study shows lower LNY in TD-positive patients, which is consistent with other studies [27] [28]. This may be partially attributed to the older age and higher rectal tumor incidence which are factors associated with fewer LNY [29]. Current research links LNY to tumor immune response status [30] [31], while a new study indicate that TD exhibits immunosuppression and immune evasion [32], further research is needed to explore potential biological links. It is noteworthy that T4 and N2 are established adverse prognostic factors. The IDEA study confirmed the necessity of risk stratification based on T and N in stage III colon cancer, we have further incorporated rectal cancer patients into our analysis, significant survival differences validate its rationality to some extent.

After risk stratification, TD was associated with age, tumor location (rectum > left colon > right colon), and fewer lymph node yield in low-risk group, this may be explained by differences in LNM, with rectal cancer patients more prone to LNM [33] and left-sided colon cancer posing a higher risk than right-sided [34]. In the high-risk group, TD was associated with perineural invasion (P = 0.005) and higher preoperative CEA (P = 0.002), indicating the association with more aggressive pathological features. Moreover, ≥3TD patients showed poorer CSS (P = 0.02; Fig. 4B) and DFS (P < 0.001; Fig. 4E) vs 1-2TD, while 1-2TD vs no-TD showed no significant difference in low-risk group. In high-risk, 1-2TD and ≥3TD had worse prognosis than no-TD (P < 0.05; Fig. 4C,F), but no significant difference between them, aligning with the conclusion reached by Nagayoshi et al. [35] that the prognosis of N2 patients is poor regardless of TD status. However, another study found that in the SEER cohort, N2 patients with ≥5 TD had a worse prognosis, though the results from their cohort did not support this conclusion [36]. Multivariate Cox regression analysis confirmed our findings, indicating increased cancer-specific mortality (HR = 2.909, 95%CI = 1.017–8.316, P = 0.046; Table 3) and recurrence risks (HR = 2.616, 95%CI = 1.408–4.861, P = 0.002; Table 3) in ≥3TD patients vs. 1-2TD in the low-risk group, while in the high-risk group, no significant survival difference was noted between 1-2TD and ≥3TD patients. We speculate that there are few patients with numerous TD in clinical practice (only 8.5 % and 3.8 % of TD-positive patients in the two cohorts have >5TD, respectively), and for patients with N2 or T4 stage, numerous LNM and deeper tumor invasion might weaken the prognostic value of TD.

The impact of TD on post-chemotherapy survival has been inconsistent in previous studies. Prior study reported that chemotherapy is independently associated with better prognosis in TD-positive CRC patients [28]. Additionally, the presence of TD did not affect the benefits of chemotherapy in stage III colon cancer [37]. However, some studies showed no survival benefit for TD-positive patients after chemotherapy [38]. We aimed to further clarify the role of TD count in the prognosis of stage III CRC patients receiving adjuvant chemotherapy. Multivariate Cox analysis revealed that chemotherapy reduced cancer-specific mortality risk by 63.5 % (HR = 0.365, 95%CI = 0.205–0.651, P = 0.001) and recurrence risk by 34.2 % (HR = 0.658, 95%CI = 0.436–0.995, P = 0.047) in all patients. Notably, patients with 1-2TD and ≥3TD both exhibited significant reduced cancer-specific mortality from chemotherapy, however, whether there were few or numerous TD, there was no benefit of chemotherapy in terms of recurrence risk. To our knowledge, there are limited studies exploring the prognostic significance of TD in relation to both T and N staging. We determined 2TD as the optimal prognostic cutoff for stage III CRC, with ≥3TD indicating poorer prognosis. TD count's prognostic value varies by risk level; in low-risk patients, ≥3TD significantly impacts survival, whereas in high-risk patients the prognostic outcomes of ≥3TD and 1-2TD are similar, which indicating that as T and N stages advance, TD count's prognostic significance diminishes. Considering TD count in low-risk patients' assessments is crucial, particularly for ≥3TD. For advanced stages, prioritizing T and N stages with TD presence as a secondary indicator may be more practical.

There are some limitations to our study. Firstly, its single-center retrospective design with a moderate sample size may yield selection bias, necessitating validation in larger, multi-center trials. Secondly, the relatively short follow-up time demands longer-term survival analysis. Lastly, we did not assess chemotherapy regimens or courses. Further exploration into the role of TD count in personalizing or improving chemotherapy regimens and courses is needed.

## Conclusion

TD indicates poor prognosis in stage III CRC, with ≥3 TD significantly worsening survival, yet the prognosis remains poor in TD-positive patients with high-risk (T4 or N2) regardless of TD count. Moreover, TD count does not influence chemotherapy's mortality benefit.

## CRediT authorship contribution statement

**Chenxiao Zheng:** Data curation. **Lingsha Xu:** Formal analysis, Data curation. **Binbin Ou:** Formal analysis. **Mohamed Bakour Abdourahaman Ibrahim:** Formal analysis. **Xuanqin Chen:** Writing – original draft. **Hangjia Xu:** Writing – original draft. **Yating Zheng:** Writing – review & editing. **Yifei Pan:** Conceptualization.

## Consent for publication

Not applicable.

## Ethics approval and consent to participate

The study was certified by the Ethics Committee of The First Affiliated Hospital of Wenzhou Medical University (KY2022–183). Given the retrospective nature of the study, informed consent was waived.

## Funding

This study is funded by Wenzhou Science and Technology Public Project (NO. Y20220184) and Horizontal Subject (50026266).

## Declaration of competing interest

The authors declare that they have no competing interests.

## Data Availability

Publicly available datasets are available from: https://seer.cancer.gov/. The supporting data of this study is available from the corresponding author upon reasonable request.

## Appendix A Supplementary data

Supplementary material Image 1
