# Supplementary material for: Prognostic significance of tumor deposit counts in stage III colorectal cancer based on T/N staging and chemotherapy status: A retrospective cohort study
Source: Surg Open Sci. 2025 Feb 22;24:42–50. doi: 10.1016/j.sopen.2025.02.004 (PMC11908539; doi:10.1016/j.sopen.2025.02.004)
Supplement: Supplementary file 1 — Supplementary material [file mmc1.docx]

Prognostic significance of Tumor Deposit Counts in Stage III Colorectal Cancer Based on T/N Staging and Chemotherapy Status: A Retrospective Cohort Study

Supplementary Material

Supplementary Table 1：Multivariate Cox analysis of the CSS for stage III CRC patients with different risk levels.

| **Variable** | **ALL** | | | **Low Risk** | | **High Risk** | |
| --- | --- | --- | --- | --- | --- | --- | --- |
|  | **HR（95%CI）** | **P-value*** | | **HR（95%CI）** | **P-value*** | **HR（95%CI）** | **P-value*** |
| **Age** |  |  |  | |  |  |  |
| ≤65 | 1 |  | 1 | |  |  |  |
| >65 | 1.954(1.042-3.665) | **0.037** | 3.673(1.436-9.393) | | **0.007** |  |  |
| **Primary site** |  |  |  | |  |  |  |
| Right colon |  |  | 1 | |  |  |  |
| Left colon |  |  | 0.812(0.257-2.566) | | 0.723 |  |  |
| Rectum |  |  | 2.590(1.134-5.915) | | **0.024** |  |  |
| **Tumor size** |  |  |  | |  |  |  |
| ≦4cm | 1 |  |  | |  |  |  |
| >4cm | 1.876(1.084-3.246) | **0.025** |  | |  |  |  |
| **Tumor Deposits** |  |  |  | |  |  |  |
| 0 | 0.854（0.460-1.587） | 0.618 | 1.063（0.463-2.440） | | 0.885 | 0.292(0.115-0.741) | **0.010** |
| 1-2 | 1 |  | 1 | |  | 1 |  |
| ≧3 | 2.351（1.106-4.996） | **0.026** | 2.909（1.017-8.316） | | **0.046** | 1.500(0.499-4.507) | 0.471 |
| **Vascular invasion** |  |  |  | |  |  |  |
| Negative | 1 |  |  | |  |  |  |
| Positive | 1.605(0.334-7.715) | 0.555 |  | |  |  |  |
| **Perineural invasion** |  |  |  | |  |  |  |
| Negative | 1 |  | 1 | |  |  |  |
| Positive | 2.576(1.309-5.072) | **0.006** | 3.059(1.290-7.254) | | **0.011** |  |  |
| **Chemotherapy** |  |  |  | |  |  |  |
| No | 1 |  | 1 | |  | 1 |  |
| Yes | 0.365(0.205-0.651) | **0.001** | 0.405(0.192-0.856) | | **0.018** | 0.218（0.194-0.506） | **0.001** |
| **Preoperative CEA** |  |  |  | |  |  |  |
| Negative | 1 |  | 1 | |  |  |  |
| Positive | 1.349(0.778-2.339) | 0.286 | 2.358（1.102-6.042） | | **0.027** |  |  |

*Pearson's Chi-squared test. The P-value for significance was < 0.05

Supplementary Table 2：Multivariate Cox analysis of the DFS for stage III CRC patients with different risk levels.

| **Variable** | **ALL** | | | **Low Risk** | | **High Risk** | |
| --- | --- | --- | --- | --- | --- | --- | --- |
|  | **HR（95%CI）** | **P-value*** | | **HR（95%CI）** | **P-value*** | **HR（95%CI）** | **P-value*** |
| **Age** |  |  |  | |  |  |  |
| ≤65 | 1 |  |  | |  |  |  |
| >65 | 1.261（0.884-1.800） | 0.201 |  | |  |  |  |
| **Primary site** |  |  |  | |  |  |  |
| Right colon | 1 |  |  | |  |  |  |
| Left colon | 1.377（0.784-2.419） | 0.266 |  | |  |  |  |
| Rectum | 2.018（1.823-1.110） | **0.030** |  | |  |  |  |
| **Tumor differentiation** |  |  |  | |  |  |  |
| Poor | 1 |  | 1 | |  |  |  |
| Moderate | 0.543（0.331-0.891） | **0.016** | 0.415（0.213-0.807） | | **0.010** |  |  |
| Well | 0.754（0.406-1.401） | 0.372 | 0.540（0.249-1.173） | | 0.119 |  |  |
| **Tumor Deposits** |  |  |  | |  |  |  |
| 0 | 0.668（0.450-0.992） | **0.045** | 0.794（0.493-1.278） | | 0.343 | 0.421（0.210-0.845） | **0.015** |
| 1-2 | 1 |  | 1 | |  | 1 |  |
| ≧3 | 1.823（1.110-2.996） | **0.018** | 2.616（1.408-4.861） | | **0.002** | 1.356（0.610-3.103） | 0.455 |
| **Lymph node yield** |  |  |  | |  |  |  |
| <12 | 1 |  | 1 | |  |  |  |
| ≧12 | 1.289（0.854-1.946） | 0.227 | 1.274（0.778-2.086） | | 0.336 |  |  |
| **Perineural invasion** |  |  |  | |  |  |  |
| Negative | 1 |  | 1 | |  | 1 |  |
| Positive | 1.616（1.099-2.374） | **0.015** | 1.380（0.885-2.153） | | 0.156 | 0.942(0.139-6.386) | 0.951 |
| Unknow | 2.186（0.847-5.643） | 0.106 | 2.337（0.700-7.805） | | 0.168 | 1.855(0.304-11.306) | 0.503 |
| **Chemotherapy** |  |  |  | |  |  |  |
| No | 1 |  | 1 | |  | 1 |  |
| Yes | 0.658（0.436-0.995） | **0.047** | 0.690（0.414-1.151） | | 0.155 | 0.515(0.262-0.969) | **0.023** |
| **Preoperative CEA** |  |  |  | |  |  |  |
| Negative | 1 |  | 1 | |  |  |  |
| Positive | 1.739（1.220-2.479） | **0.002** | 1.752（1.118-2.746） | | **0.014** |  |  |
| **PreoperativeCA19-9** |  |  |  | |  |  |  |
| Negative |  |  | 1 | |  | 1 |  |
| Positive |  |  | 1.517（0.863-2.666） | | 0.147 | 0.983(0.460-2.102) | 0.965 |
| *Pearson's Chi-squared test. The P-value for significance was < 0.05 | | | | | | |  |

Supplementary Table 3: Multivariate Cox analysis of the CSS for stage III CRC patients with varying TD counts.

|  | **0TD** | | | **1-2TD** | | **≥3TD** | |
| --- | --- | --- | --- | --- | --- | --- | --- |
| **Variable** | **HR（95%CI）** | **P-value*** | | **HR（95%CI）** | **P-value*** | **HR（95%CI）** | **P-value*** |
| **Age** |  |  |  | |  |  |  |
| ≤65 | 1 |  |  | |  |  |  |
| >65 | 0.347(0.138-0.870) | 0.073 |  | |  |  |  |
| **Primary site** |  |  |  | |  |  |  |
| Right colon | 1 |  |  | |  |  |  |
| Left colon  Rectum | 1.871（0.518-6.762）  1.982（0.940-3.655） | 0.339  0.062 |  | |  |  |  |
| **Risk stratification** |  |  |  | |  |  |  |
| Low risk |  |  | 1 | |  |  |  |
| High risk |  |  | 3.433(1.345-8.759) | | **0.010** |  |  |
| **Lymph node yield** |  |  |  | |  |  |  |
| <12 |  |  |  | |  |  |  |
| ≧12 |  |  |  | |  |  |  |
| **Vascular invasion** |  |  |  | |  |  |  |
| Negative |  |  | 1 | |  | 1 |  |
| Positive |  |  | 1.767(0.578-5.399) | | 0.318 | 4.015(0.841-19.171) | 0.081 |
| Unknow |  |  |  | |  |  |  |
| **Perineural invasion** |  |  |  | |  |  |  |
| Negative | 1 |  |  | |  |  |  |
| Positive | 3.846(1.290-9.427) | **0.016** |  | |  |  |  |
| **Chemotherapy** |  |  |  | |  |  |  |
| No | 1 |  | 1 | |  | 1 |  |
| Yes | 0.350(0.146-0.837) | **0.018** | 0.347(0.138-0.870) | | **0.024** | 0.272(0.077-0.960) | **0.040** |
| **Preoperative CEA** |  |  |  | |  |  |  |
| Negative |  |  |  | |  |  |  |
| Positive |  |  |  | |  |  |  |
| *Pearson's Chi-squared test. The P-value for significance was < 0.05. | | | | | | |  |

Supplementary Table 4: Multivariate Cox analysis of the DFS for stage III CRC patients with varying TD counts.

|  | **0TD** | | | **1-2TD** | | **≥3TD** | |
| --- | --- | --- | --- | --- | --- | --- | --- |
| **Variable** | **HR（95%CI）** | **P-value*** | | **HR（95%CI）** | **P-value*** | **HR（95%CI）** | **P-value*** |
| **Gender** |  |  |  | |  |  |  |
| Male |  |  | 1 | |  |  |  |
| Female |  |  | 1.997（1.112-3.587） | | **0.021** |  |  |
| **Primary site** |  |  |  | |  |  |  |
| Right colon |  |  |  | |  | 1 |  |
| Left colon |  |  |  | |  | 0.180（0.041-0.787） | **0.023** |
| Rectum |  |  |  | |  | 0.170（0.037-0.776） | **0.022** |
| **Tumor size** |  |  |  | |  |  |  |
| ≦4cm |  |  |  | |  | 1 |  |
| >4cm |  |  |  | |  | 1.554（0.656-3.679） | 0.316 |
| **Risk stratification** |  |  |  | |  |  |  |
| Low risk |  |  | 1 | |  |  |  |
| High risk |  |  | 2.725（1.439-5.162） | | **0.002** |  |  |
| **Lymph node yield** |  |  |  | |  |  |  |
| <12 |  |  | 1 | |  |  |  |
| ≧12 |  |  | 0.518（0.276-0.972） | | **0.040** |  |  |
| **Vascular invasion** |  |  |  | |  |  |  |
| Negative | 1 |  | 1 | |  |  |  |
| Positive | 1.631（0.816-3.259） | 0.166 | 1.585（0.844-2.974） | | 0.152 |  |  |
| **Perineural invasion** |  |  |  | |  |  |  |
| Negative | 1 |  |  | |  |  |  |
| Positive | 2.119（1.190-3.774） | **0.011** |  | |  |  |  |
| **Chemotherapy** |  |  |  | |  |  |  |
| No | 1 |  | 1 | |  | 1 |  |
| Yes | 0.434（0.211-0.891） | **0.023** | 0.638(0.332-1.224) | | 0.177 | 0.422(0.173-1.031) | 0.058 |
| **Preoperative CEA** |  |  |  | |  |  |  |
| Negative | 1 |  |  | |  |  |  |
| Positive | 1.757（1.0380-2.975） | **0.036** |  | |  |  |  |
| *Pearson's Chi-squared test. The P-value for significance was < 0.05 | | | | | | |  |

Supplementary Table 5: Multivariate Cox analysis of the CSS and DFS in TD positive stage III CRC patients with different risk levels and TD count (1-2TD group as reference).

|  |  | **All** | | **Low risk** | | **High risk** | |
| --- | --- | --- | --- | --- | --- | --- | --- |
| **Survival** | **TD** | **HR（95%CI）** | **P** | **HR（95%CI）** | **P** | **HR（95%CI）** | **P** |
| **CSS** | **1-2 TD** | 1 |  | 1 |  | 1 |  |
|  | **≥3 TD** | 2.351（1.106-4.996） | 0.026 | 3.445 (1.254-9.465) | 0.017 | 1.624 (0.442-5.927) | 0.466 |
| **DFS** | **1-2 TD** | 1 |  | 1 |  | 1 |  |
|  | **≥3 TD** | 1.808 (1.073-3.039) | 0.027 | 1.934 (1.095-3.416) | 0 .024 | 1.645 (0.694-3.885) | 0.260 |

Supplementary figure1: Survival curves of patients with stage III rectal cancer, stratified by risk level: (A) CSS of patients with rectal cancer; (B) DFS of patients with rectal cancer.


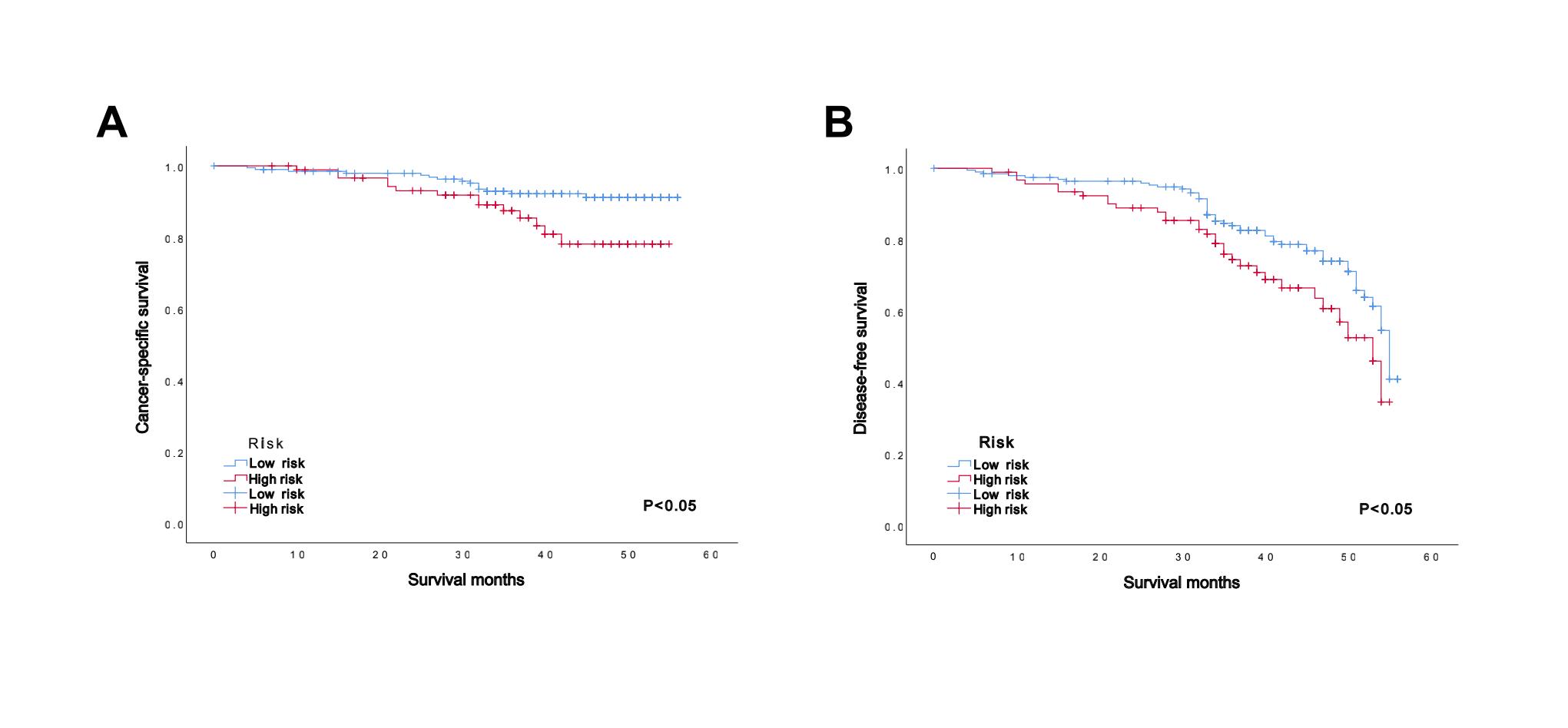


Supplementary figure2: X-tile analysis determines the optimal cutoff value for the number of TD in CSS.
